# ZDQ-0620, a Novel Phosphatidylinositol 3-Kinase Inhibitor, Inhibits Colorectal Carcinoma Cell Proliferation and Suppresses Angiogenesis by Attenuating PI3K/AKT/mTOR Pathway

**DOI:** 10.3389/fonc.2022.848952

**Published:** 2022-03-02

**Authors:** Xiaochun Qin, Mingyue Liu, Chang Xu, Bo Xing, Xiangbo Xu, Yuting Wu, Huaiwei Ding, Qingchun Zhao

**Affiliations:** ^1^ Department of Pharmacy, General Hospital of Northern Theater Command, Shenyang, China; ^2^ Department of Life Science and Biochemistry, Shenyang Pharmaceutical University, Shenyang, China; ^3^ Key Laboratory of Structure-Based Drug Design and Discovery of Ministry of Education, Shenyang Pharmaceutical University, Shenyang, China

**Keywords:** colorectal cancer, cell cycle arrest, apoptosis, angiogenesis, PI3K

## Abstract

The PI3K/AKT pathway plays a central role in human cancers, aberrant activation of this pathway is associated with tumorigenesis, cancer progression and angiogenesis. Based on the importance of the PI3K/AKT pathway in malignancies, we developed a 4-aminoquinazoline derivative, ZDQ-0620, initially envisioned as a novel pan-PI3K inhibitor. This study aimed to evaluate the potential target of ZDQ-0620 and its anticancer effect in human colorectal carcinoma (CRC). PI3K-kinase activity test showed IC50 of ZDQ-0620 against PI3Ka was 0.5 nM; molecular docking, CETSA assay and western blotting was further performed to predict ZDQ-0620 was a PI3K/AKT pathway inhibitor by targeting PI3K. To identify the effect of ZDQ-0620 on CRC cells, Sulforhodamine B (SRB) assay, flow cytometry, and Cell morphology analysis were conducted. The results showed that ZDQ-0620 inhibited the proliferation, migration and invasion of CRC cells, induced apoptosis through G0/G1 cell cycle arrest and mitochondrial pathway. Additionally, ZDQ-0620 inhibited the migration and tube formation of human umbilical vein endothelial cells (HUVECs). *In vivo*, neovascularization of rat aortic ring and chick chorioallantoic membrane (CAM) induced by VEGF was diminished when treated with ZDQ-0620. These results indicate that ZDQ-0620 induce apoptosis and anti-angiogenesis *via* inhibits the PI3K/AKT pathway. We suggest that the great potential of ZDQ-0620 as an effective treatment candidate against CRC.

## Introduction

Colorectal cancer, one of the most common cancers worldwide, has been a serious threat to human health (1). There are about 1.36 million new cases of colorectal cancer in the world every year, which is the third most common malignant tumor globally (2). The incidence of colorectal cancer ranks the third in men and the second in women. Every year about 690,000 cases of death, ranking the fourth malignancy (3). Despite advances in diagnosis and treatment over the past few decades, colorectal cancer remains a major health problem and a significant socio-economic burden (4). Only a few cases of CRC are detected early enough, so early accurate diagnosis and targeted treatment plan is particularly important, such as surgery, chemotherapy, and radiotherapy (5, 6). Rapid tumor growth, characterized by rapid progression and poor prognosis, is a major problem affecting the treatment of CRC (7).

With the research on oncogenic signaling pathways that regulate the proliferation, invasion, metastasis and angiogenesis of cancer cells, several possible hot therapeutic targets have been identified recently (8, 9). Among them, the phosphoinositide 3-kinase (PI3K)/AKT/mTOR pathway is one of the most frequently activated in human cancers (10). PI3K/AKT/mTOR pathway is widely present in cells and is involved in cell growth, proliferation, differentiation regulation and other aspects (11). PI3K phosphorylates PIP2 to produce the second messenger phosphatidylinositol-3,4,5 -trisphosphate (PIP3) (12). Overactivation of PI3K leads to an increase in PIP3 levels, which in turn activates downstream AKT phosphorylation (13). In addition, overexpression of AKT has been proved in many cancers including CRC, which has a variety of biological activities, including inhibition of tumor cell apoptosis, promotion of invasion and metastasis, and regulation of tumor angiogenesis (8, 14). MTOR, a serine/threonine protein kinase, is a downstream molecule of AKT in the PI3K/AKT pathway and is involved in the regulation of protein synthesis, cell apoptosis, angiogenesis, etc (15). Activated PI3K/AKT can further activate mTOR through the TSC1/2 complex. Subsequent activation of mTOR promotes cell growth and cell cycle progression through phosphorylated translation regulator p70S6 kinase (p70S6K) and eukaryotic promoter (EIF) 4E binding protein 1 (4EBP1) (16, 17). Therefore, PI3K/AKT/mTOR pathway has become a key therapeutic target for cancer treatment (10, 11). Inhibiting the overactivation of the PI3K pathway in CRC appears to be a promising therapeutic strategy.

**Figure 1:**
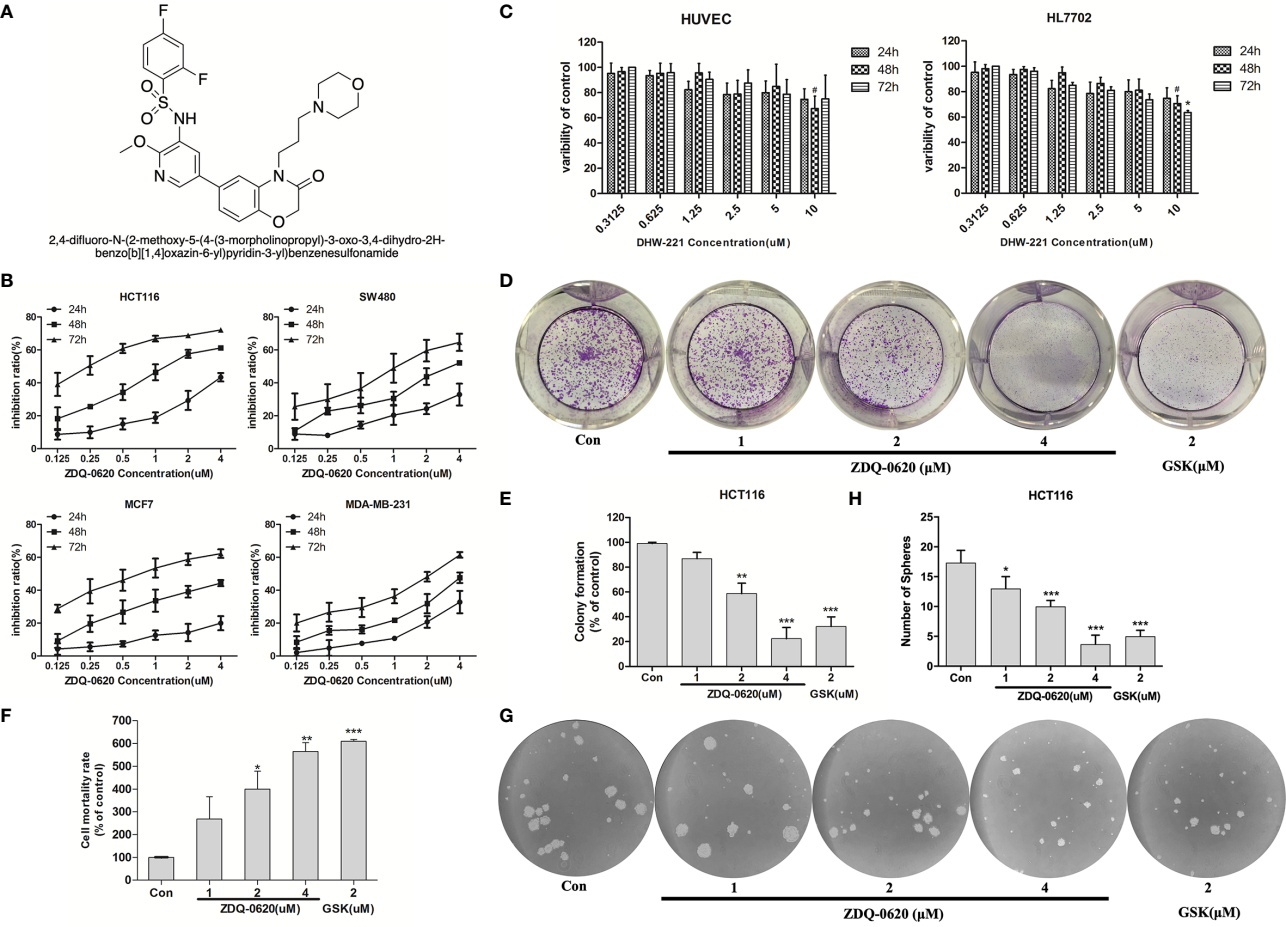
ZDQ-0620 inhibits the viability and proliferative ability of carcinoma cells. **(A)** The structure of compound ZDQ-0620. **(B)** The viability of HCT116, SW-480, MCF-7, and MDA-MB-231 cells treated with various concentrations of ZDQ-0620 at 24, 48, and 72 h was determined by sulforhodamine B (SRB) assay. **(C)** A SRB assay was also used to determine the cytotoxicity of ZDQ-0620 toward normal cell lines (HUVEC and HL7702) at 72). **(D**–**H)** The inhibitory effect of ZDQ-0620 on the growth of HCT116 cells was measured by a colony formation assay **(D)**, LDH assay **(F)**, and tumor sphere formation assay **(G)**. Panel **(E)** the quantitative results of the colony formation assay. Panel **(H)** the quantitative results of the tumor sphere formation assay. Each value is the mean (± SD) from triplicate samples; ^#^ *p* < 0.05 vs. the control; *p < 0.05, **p < 0.01, ***p < 0.001 *vs*. the control. One-way analysis of variance followed by Tukey’s *post hoc* multiple-comparisons test.

To find a novel structure-like PI3K inhibitor, we initiated a pharmacophore-oriented design. Our previous study designed and synthesized a series of 4-aminoquinazolines derivatives containing hydrophilic group acting on the PI3K/AKT/mTOR pathway (18). Among them, ZDQ-0620 (2,4-difluoro-N-(2-methoxy-5-(4-(3-morpholinopropyl)-3-oxo-3,4-dihydro-2H-benzo[b][1,4]oxazin-6-yl)pyridin-3-yl)benzenesulfonamide) (**Figure 1A**) exhibited the best *in vitro* anti-cellular activity. In the present study, we intend to characterize biological effect of ZDQ-0620 in CRC cells, including cell proliferation, migration, invasion and angiogenesis, and further clarify the underlying mechanisms through modulating the PI3K/AKT/mTOR pathway, which sheds a light upon the thought of developing PI3K inhibitor and provides a promising direction as an anti-cancer drug against CRC in future.

## Materials and Methods

### Cells and Reagents

All human cell lines used in this study were purchased from ATCC (Manassas, VA) and were cultured in RPMI-1640 medium (Gibco, USA), containing 10% fetal bovine serum (Hyclone, USA), penicillin-streptomycin (100 U/ml, Hyclone) at 37°C in a 5% CO_2_ incubator. Propidium iodide (PI), Sulforhodamine B (SRB), and Hoechst 33342 were purchased from Sigma–Aldrich (St. Louis, MO). Primary antibodies aganist p-AKT (Ser473) (#9271), p-AKT (Thr308) (#9275), AKT (#4691), p-mTOR (#2971), mTOR (#2972), 4EBP1 (#9644), p-4EBP1 (#2855), p70S6K (#9202), p-p70S6K (#9205), Cyclin D1 (#2922), Bcl-2 (#4223), Bax (#5023), Caspase-3 (#9662), Cleaved caspase-3 (#9661), Caspase-9 (#9508), Cleaved caspase-9 (#7237), PARP (#9532), and β-actin (#3700) were purchased from Cell Signaling Technology (MA, USA). HRP and FITC-conjugated secondary antibodies were obtained from ZSGB-BIO (Beijing, China). All reagents were stored at -80°C.

### Measurement of Cell Proliferation

#### Sulforhodamine B (SRB) Assay

Cells were inoculated into 96-well microplates at a concentration of 200 μL/well. Different concentrations of ZDQ-0620 were added for 24h, 48h and 72h, respectively, and fixed with 50% trichloroacetic acid solution for 1 hour. After washing with deionized water for 5 times, 0.4% sulforhodamine B (SRB) was used for staining in the darkroom. After 30min, the residual SRB was removed with 1% glacial acetic acid, and dried at room temperature. Finally, the absorbance was measured at 490 nm with a microplate analyzer (Synergy 2, Bio-Tek, USA).

#### Colony Formation Assay

The cells were seeded in 6-well plates at a density of 2×10^3^ cells per well and cultured overnight. Cells were then treated with specified concentrations of ZDQ-0620, GSK2126458 or DMSO for 48h. After replacing the new medium, the cells were cultured for 7 days. The culture medium was discarded, fixed with 4% paraformaldehyde for 10 min, stained with 0.1% crystal violet for 15 min, and photographed with digital camera.

#### LDH Assay

Cells were inoculated in 96-well plates at 3×10^3^ and co-cultured with specific concentrations of ZDQ-0620, GSK2126458 or DMSO for 48 h. The supernatant was collected and co-incubated according to kit instructions (Wanlei, China), and the absorbance was determined at 490 nm using an enzyme plate analyzer (Synergy 2, Bio-Tek, USA). The percentage of lactate dehydrogenase release can be used as an indicator of cytotoxicity of compounds.

#### Tumor Sphere Formation Assay

Cells were inoculated at 4×10^3^ on a low-attached 6-well plate (Corning, USA) and cultured in serum-free DMeM-F12 (Gibco) containing EGF (20ng/ml, Peprotech), B-FGF (20ng/ml, Peprotech) and B27(1:50 dilution, BD Biosciences). After 9 days, the number of tumor globules formed was counted by phase contrast microscope.

### Determination of Apoptosis

#### Cell Morphology Analysis

Cells with a density of 2×10^4^ were cultured overnight on a 6-well plate. After 48h treatment with ZDQ-0620, GSK2126458 or DMSO, Hoechst 33342 (Beyotime Bio, China) was stained at 37°C for 20 min. After two PBS rinses, fluorescence microscopy (IX71, Olympus, Japan) was used to take pictures. The nuclei of apoptotic cells are densely stained, completely or partially bright blue.

#### Annexin V-FITC/PI *Assay*

In brief, the cells were inoculated in 6-well plates at a density of 2 × 10^3^ per well. ZDQ-0620, GSK2126458 or DMSO were treated for 48 h, and the collected cells were stained in the dark with Annexin V-FITC and PI (Wanlei, China) for 10-20 min. Finally, the cells were sorted and quantitatively analyzed by flow cytometry (Becton-Dickinson, NJ, USA).

#### Transmission Electron Microscopy

Cells were seeded into 6-well plates at a density of 5 × 10^4^ cells/well. After treated with ZDQ-0620 for 24 h, the cells were collected and fixed with 3% glutaraldehyde, then fixed with 1% O_S_O_4_, then dehydrated with ethanol step by step, embedded and sected. It was stained with uranium acetate and lead citrate and observed under transmission electron microscope. (H-7650, Hitachi, Japan).

#### Mitochondrial Membrane Potential Assay (△ψm)

The cells were inoculated in 6-well plates at a density of 1× 10^4^ overnight and treated with the specified concentration of ZDQ-0620, GSK2126458 or DMSO for 48 h. The cells were incubated in an incubator at 37°C for 20 min with 1 mL of JC-1 staining solution per well. After washing with PBS, the cells were imaged under a fluorescence microscope (IX71, Olympus, Japan). Red fluorescence indicates normal MMP, while green fluorescence indicates decreased MMP.

### PI3K Activity Assay

The effect of ZDQ-0620 on PI3K kinase assay was measured by ADP-GloTM Lipid Kinase Systems (Promega, USA). Treatment for experiments was carried out according to the manufacturer’s instructions.

### Cell Cycle Assay

Cells were seeded in 6-well plates at a density of 2 × 10^5^ per well. After treated with ZDQ-0620 for 48 hours, cells were collected and fixed overnight in 70% cold ethanol at 20°C. The cells were rinsed with cold PBS buffer and then stained darkly with PI for 15 minutes at room temperature. The samples were analyzed by FACS Calibur flow cytometry (Becton-Dickinson, NJ, USA).

### Western Blotting

Cells were exposed to gradient dose of ZDQ-0620 for 48 h. The expression levels of related proteins in cells were determined by standard Western blot.

### Molecular Docking

Using the GLIDE module of the Schrodinger software with the Maestro interface to predict the binding modes of PI3K and ZDQ-0620. ZDQ-0620 was drawn by ChemBio3D Ultra 13.0 software and prepared using the Ligprep. Then, the crystal structures of PI3Kα was download from the Protein Data Bank and prepared by the Protein Preparation Wizzard. The active pocket of proteins was generated with the Grid Generation tool. Other parameters are keep as software defaults. The docking poses were analyzed with Pymol.

### Immunofluorescence Images

Cells with a density of 1×10^4^ were inoculated overnight in 6-well plates and treated with ZDQ-0620 (2μM) for 24 h, fixed with 4% paraformaldehyde, permeated with 0.2%Trion-X for 20min, incubated with immunostain blocking buffer for 1 h, and incubated overnight with p-Akt (Ser473) at 4°C. After washing with 4 °C of PBS, the cells were incubated at room temperature for 1 h with secondary antibody, and then the nuclei were stained with DAPI for 15min. The staining cells were imaged by fluorescence microscopy (IX71, Olympus, Japan).

### CETSA Assay

HCT116 cells were treated with a specified concentration of ZDQ-0620 for 3 h, then the cells were collected with PBS containing protease inhibitors and transferred into 200μL EP tubes. Cells were placed in a PCR apparatus (2720, Gene, Singapore) and subjected to heat shock at 37~77°C for 3min. Lysed for 3 times in liquid nitrogen - room temperature cycles. Centrifuge at 13 000 RPM at 4°C for 20 min. Loading buffer was added and standard Western blot was performed.

### Wound Healing Scratch Assay

Cells of 1× 10^4^ density were inoculated in 6-well plates until 90% were full. Cell-free area was formed in the designated area of sterile pipetting tip, and cells were treated with ZDQ-0620, GSK2126458 or DMSO after twice washing with PBS. Images were taken under an inverted microscope (IX71, Olympus, Japan) at 0 and 24 h, and the Image J software analyzed and calculated the proportion of cells migrating to the cleared area (wound healing). Migration rate=(1-width _24h_)/width _0h_.

### Migration and Invasion Assay

Cells with a density of 4 × 10^4^ were seeded into the upper cavity of a 24-well transwell plate (Corning, USA) without or coated with matrix gel (Becton Dickinson, USA). The lower cavity was cultured in complete medium containing 10% fetal bovine serum for 48h. The migrated and invaded cells were fixed with 70% acetaldehyde, stained with 0.1% crystal violet and photographed under a microscope (IX71, Olympus, Japan).

### Tube Formation Assay

The matrix adhesive was mixed with RPMI-1640 medium in equal volume, and then added into 48-well plate at 100μL/well to cure. 1 × 10^5^ cells/100μL/well HUVEC cells were seeded into 48-well plates with the specified concentration of ZDQ-0620, GSK2126458 or DMSO. After culture for 3 h, the tube formation was observed by inverted microscope (IX71, Olympus, Japan).

### Chick Chorioallantoic Membrane (CAM) Assay

The sterilized fertilized eggs (SAIS Poultry Co. Ltd., China) were incubated in an incubator at 37.8*°C* and 60% humidity for 5 days. The 4cm^2^ pore was opened at the end of the air chamber, and the eggshell membrane was discarded to protect the chorioallantoic membrane of the chicken embryo from damage. VEGF and/or ZDQ-0620 and GSK2126458 were added to allantoic membrane and sealed for further incubation for 48 h. The microvascular growth was observed with digital camera. Ten eggs in each group were incubated.

### Rat Aortic Ring Sprouting Assay

The rats were purchased from Vital River Technology Co., Ltd (Beijing, China). The aortas of 6-week-old SD rats was cut into 2mm long rings and randomly divided into groups, then the aortas were embedded in a 96-well plate with 70 μL matrix gel and solidified in an incubator for 30 min. The specified concentrations of VEGF and/or ZDQ-0620 and GSK2126458 were added and incubated for 7 days. Photograph with a microscope (Olympus, Tokyo, Japan).

### Statistical Analysis

Unless otherwise stated, all experiments in this study were repeated three times. Statistical analysis was performed using GraphPad Prism 5 (GraphPad software, CA, USA). Groups were compared with the unpaired Student’s t-tests, and multiple groups were compared with the one-way ANOVA. All data were expressed as means values ± SD. p < 0.05 indicated statistically significant.

## Results

### Effect of ZDQ-0620 on the Growth of HCT116 Cells

To verify the function of ZDQ-0620 as a new therapeutic compound, we first measured cell growth inhibition on four cancer cell lines (HCT116, SW480, MCF-7, MDA-MB-231) using SRB assay. ZDQ-0620 significantly inhibited the growth of all tested cells in a dose- and time-dependent manner (**Figure 1B**). Especially, 0.1uM of ZDQ-0620 induced strong reduction in growth rate of HCT116 reached 40% at 72h. To predict side effects of ZDQ-0620, it was exposed to normal cell lines (**Figure 1C**). The cytotoxicity of ZDQ-0620 to HUVEC and HL7702 cells was much lower than that to tumor cells, suggesting the hypo-toxicity of ZDQ-0620 to normal cells.

Moreover, HCT116 cells were selected to investigate the long-term toxic effect of ZDQ-0620 on the proliferation. The colony formation, LDH release and Sphere-forming of HCT116 cells were significantly inhibited in a dose-dependent manner (**Figures 1D–H**), the activity value was stronger than that of GSK2126458, as a positive drug. These results suggested that ZDQ-0620 significantly inhibited the growth of cancer cells and has lower cytotoxicity in normal cell lines.

### ZDQ-0620 Induces G0/G1 Cell Cycle Arrest and Induces CRC Cells Apoptosis Through the Mitochondrial Pathway

**Figure 2:**
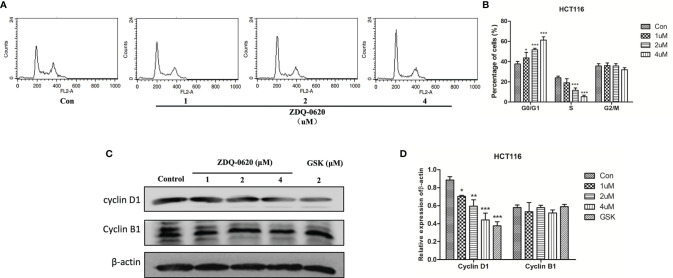
ZDQ-0620 induces HCT116 cell cycle arrest. **(A)** ZDQ-0620 were performed by PI staining, hence followed by flow cytometry (FACS) detection. **(B)** The raw histogram is shown on the left and the quantification of individual cell cycle stages on the right. **(C)** The expression levels of cell cycle-related proteins in HCT116 cells after 48 h of treatment with the specified concentrations of ZDQ-0620 and GSK2126458 were assessed by western blotting. **(D)** The histogram shows average protein expression levels. Each value is the mean ( ± SD) from triplicate samples; *p < 0.05, **p < 0.01, ***p < 0.001 *vs*. the control. One-way analysis of variance followed by Tukey’s *post hoc* multiple-comparisons test.

To examine the mechanism responsible for ZDQ-0620-mediated cell growth inhibition, the cell cycle distribution was performed by PI staining, hence followed by flow cytometry (FACS) detection. As shown in **Figures 2A, B**, ZDQ-0620 induced a dose-dependent accumulation of cells in the G0/G1 phase. Moreover, to confirmed the effects of ZDQ-0620 on cell cycle regulation, we examined the expression of endogenous cyclins after 48 h treatment with ZDQ-0620. As shown in **Figures 2C, D**, ZDQ-0620 played an important role in G0/G1 cell cycle arrest by down-regulating the levels of cyclin D1, but has little effect on cyclin B1, which regulates the G2/M phases.

**Figure 3:**
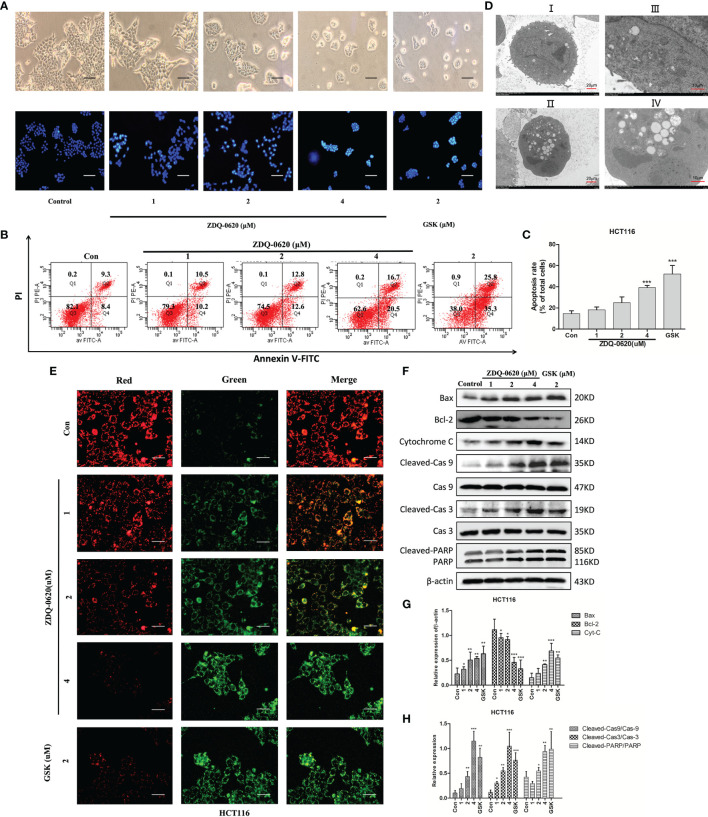
ZDQ-0620 induces HCT116 cells apoptosis. **(A)** Morphology of the cells (magnification, ×40) and Hoechst 33342 staining results. Apoptotic cells are bright blue; scale bar, 50 μm. **(B)** Analysis of the apoptotic effects of ZDQ-0620 and GSK2126458 by Annexin V/propidium iodide (PI) staining. **(C)** The histograms show the percentages of apoptotic HCT116 cells following treatment with ZDQ-0620 and GSK2126458 for 48 h (right). **(D)** Transmission electron microscopic analysis of the morphological changes occurring in cells after 48 h treatment (I and II: scale bar = 20 μm [left]; III and IV: scale bar = 10 μm [right]). **(E)** Effect of mitochondrial membrane potential detection on HCT-116. At high mitochondrial membrane potential, JC-1 aggregates in the matrix of mitochondria and forms a polymer (J-aggregates), which produces red fluorescence. At low mitochondrial membrane potential, JC-1 could not aggregate in the mitochondrial matrix, and as a monomer, JC-1 could produce green fluorescence; scale bar, 100 μm. **(F)** The expression levels of apoptosis-related proteins were detected by western blot. **(G, H)** Bar graph of the quantitative result. Each value is the mean (± SD) from triplicate samples; *p < 0.05, **p < 0.01, ***p < 0.001 *vs*. the control. One-way analysis of variance followed by Tukey’s *post hoc* multiple-comparisons test.

The PI3K/AKT/mTOR pathway can prevent programmed death of tumor cells and inhibit apoptosis, thus promoting the survival of tumor cells. To assess the pro-apoptotic effect of ZDQ-0620, inverted microscope and Hoechst 33342 staining assay was used. As shown in **Figure 3A**, apoptosis was confirmed by the presence of condensed chromatin and nuclear fragmentation. ZDQ-0620 treatment group marked with brighter fluorescence in Hoechst staining assay. In addition, cell apoptosis was detected by Annexin V/PI staining. In **Figures 3B, C**, compared with the control, the proportion of apoptotic cells (Annexin V positive) in ZDQ-0620 increased significantly and showed a concentration-dependent manner. The ultrastructure of cells observed by transmission electron microscopy was <0.2 μm, which is the gold standard for the determination of apoptosis. As shown in **Figure 3D**, cells treated with ZDQ-0620 showed typical apoptotic characteristics, such as peripheral nuclear marginalization and chromatin condensation. Pictures I and III showed control group with clear cell spacing and intercellular connections, but not tight connections. In contrast, cells treated with ZDQ-0620 showed increased heterochromatin in the nucleus, and the chromatin condensed into apoptotic bodies in pictures II and IV.

In order to further clarify the potential mechanism of ZDQ-0620-induced apoptosis, mitochondrial membrane potential was detected by JC-1 staining assay after 48 h of treatment (**Figure 3E**). As the number of apoptotic cells increased, the proportion of red fluorescence and green fluorescence decreased gradually in a dose-dependent manner. To further verify these results, western blot was used to detect the expression of apoptosis-related proteins. **Figures 3F–H** indicated that ZDQ-0620 up-regulated the expression of Bax, cytochrome C, down-regulated the expression of anti-apoptotic protein Bcl-2. The expression levels of cleaved caspase-3/9 and cleaved PARP were also significantly increased (p < 0.01). These results suggest that ZDQ-0620 could activate the caspase-dependent apoptosis cascade through mitochondrial pathway, thereby inducing apoptosis of HCT116 cells.

### ZDQ-0620 Effectively Blocks the PI3K/AKT/mTOR Signaling Pathway by Direct Targets PI3K

**Figure 4:**
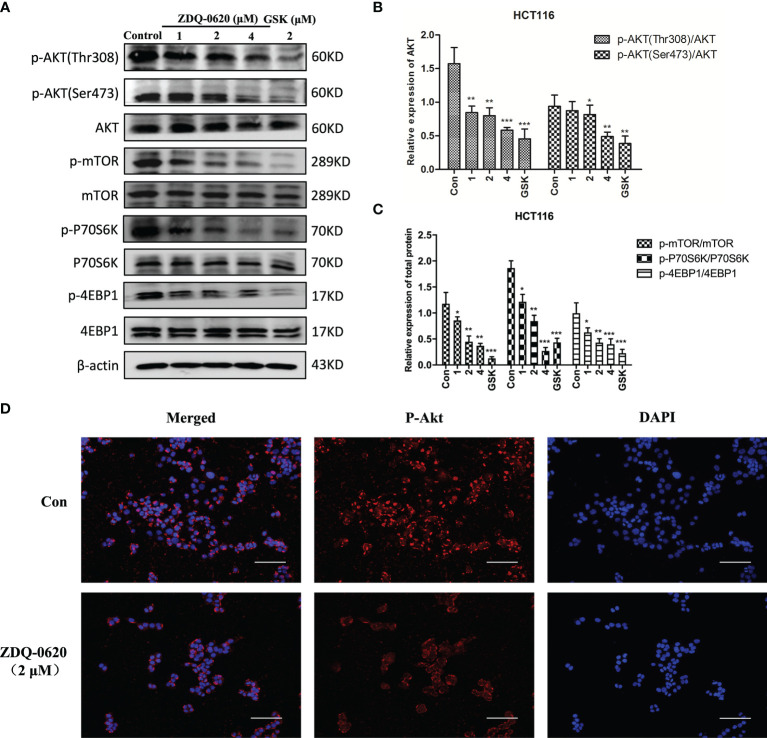
ZDQ-0620 blocks the PI3K/AKT/mTOR pathway. **(A)** HCT116 cells were treated with ZDQ-0620 or GSK2126458 for 48 h following which western blotting was performed on the cell lysates. **(B, C)** The histogram shows average protein expression levels. **(D)** Immunofluorescence imaging of p-AKT in HCT116 cells treated with ZDQ-0620. Anti-rabbit p-AKT (Ser473) antibody was used for labeling and the nuclei were stained with DAPI; scale bar, 50 μm. Each value is the mean (± SD) from triplicate samples; *p < 0.05, **p < 0.01, ***p < 0.001 *vs*. the control. One-way analysis of variance followed by Tukey’s *post hoc* multiple-comparisons test.

PI3K/mTOR and its downstream targets, 4EBP1 and P70S6K, are important factors for tumor cell survival and proliferation. To elucidate the anticancer mechanism of ZDQ-0620, western blot analysis was used evaluated the effects of ZDQ-0620 on the PI3K/AKT/mTOR pathway in HCT116. The results indicate that ZDQ-0620 could significantly suppress expression AKT and mTOR phosphorylation, which subsequently repressed phosphorylation of 4EBP1 and P70S6K (**Figure 4A**), and these effects were concentration-dependent (**Figures 4B, C**). Immunofluorescence analysis also confirmed the same results, the phosphorylation levels of AKT were significantly inhibited by ZDQ-0620 in HCT116 cells (**Figure 4D**).

**Figure 5:**
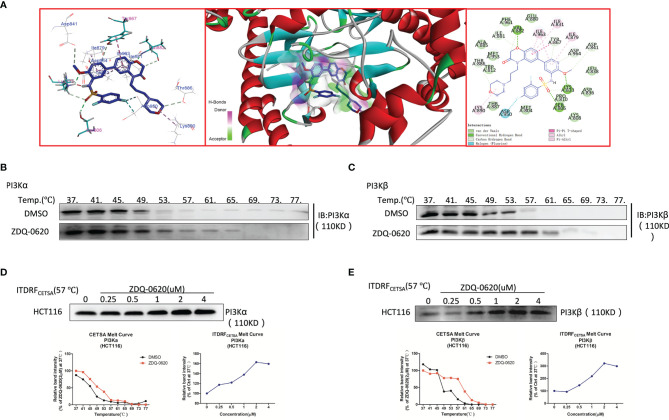
ZDQ-0620 inhibits PI3K target activity. **(A)** Predicted binding modes for ZDQ-0620 with PI3Kα (PDB code: 4JPS). **(B–E)** A cellular thermal shift assay (CETSA) and an isothermal dose-response fingerprinting (ITDRF)cetsa were used to evaluate the thermal stability of ZDQ-0620 bound to PI3Kα/β.

Then the target of the compound action was further identified. In order to explore the combination mode between ZDQ-0620 and PI3K, Maestro was used for molecular docking, and Discovery Studio 4.0 Visualizer was used to analyze the interaction mode. As shown in **Figure 5A**, Lys833 in the active pocket of PI3K forms a strong salt bridge with nitrogen on the ZDQ-0620 sulfonamide group, and forms a hydrogen bond with oxygen on the pyridine ring. In addition, the oxygen of the parent group of sulfonyl and benzooxazinone forms hydrogen bonds with Ser806 and Val882, respectively. The hydrophobic interaction between the morpholine ring and Lys890 further stabilized the binding of PI3K to ZDQ-0620. In conclusion, ZDQ-0620 can form a stable binding mode with PI3K, and the introduction of ethylmorpholine can increase the water-solubility and activity of ZDQ-0620. Then, the inhibitory activity of ZDQ-0620 against PI3 kinase was investigated. As shown in **Table 1**, ZDQ-0620 showed strong inhibitory activity against four PI3 kinase subtypes, and the inhibitory activity was below 160 nM. The inhibitory activity against PI3Kα was the best with IC50 of 0.5 nM. To further evaluate the binding character between ZDQ-0620 and PI3K, the CETSA assay was used with the treatment of HCT116 with or without ZDQ-0620. From CETSA experiments, the apparent aggregation temperatures (Tagg) were obtained with either ZDQ-0620 or DMSO, which could be compared, and substantial shifts demonstrated the binding of ZDQ-0620 and target proteins. As shown in **Figures 5B, C**, after ZDQ-0620 bound with PI3K, the thermal stabilization of PI3K were increased compared with the control groups (DMSO), and this thermal stabilization between ZDQ-0620 and PI3K was dose-dependent from ITDRF_CETSA_ (**Figures 5D, E**). In conclusion, ZDQ-0620 can effectively blocks the PI3K/AKT/mTOR signaling pathway by direct targets PI3K.

**Table 1:** IC_50_ of ZDQ-0620 against PI3K kinase *in vitro*.

Compound	PI3Kα(nM)	PI3Kβ (nM)	PI3Kγ (nM)	PI3Kδ (nM)
ZDQ-0620	0.5	93	55	152

### ZDQ-0620 Inhibits the Migration, Invasion and Interaction of HCT116 and HUVEC

**Figure 6:**
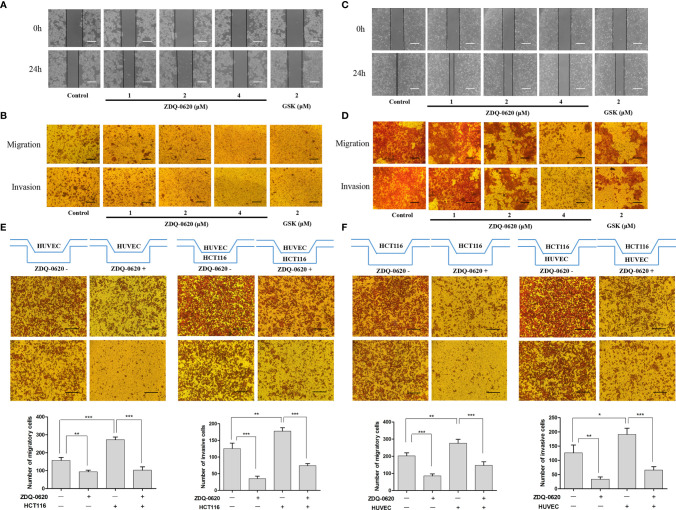
ZDQv-0620 inhibits the migration, invasion and interaction. **(A, C)** Cell migratory ability was tested using a wound healing assay; scale bar, 50 μm. **(B, D)** Cell invasiveness was assessed using a Transwell assay with Matrigel; scale bar, 50 μm. **(E)** HUVECs were inoculated alone in the upper chamber or co-cultured with HCT116 cells in the lower chamber with or without ZDQ-0620 (2uM) for comparison, Scale bar =100 µm. **(F)** HCT116 was inoculated alone in the upper chamber or co-cultured with HUVECs cells in the lower chamber with or without ZDQ-0620 (2uM) for comparison. A histogram of the invaded and migrated rates is shown at the bottom. Each value is the mean (± SD) from triplicate samples; *p < 0.05, **p < 0.01, ***p < 0.001 *vs*. the control. One-way analysis of variance followed by Tukey’s *post hoc* multiple-comparisons test.

The PI3K/AKT signaling pathway plays an important role in cancer cell migration and invasion. To determine the effects of ZDQ-0620 on colonic carcinoma cell migration and invasion ability, we first performed wound healing and invasion assays in HCT116 cell line. For wound healing and transwell migration assay, as expected, ZDQ-0620 significantly inhibited HCT116 cell metastasis in a concentration-dependent manner (**Figures 6A, B**). For transwell invasion assay, the results showed that the inhibition of invasiveness ability concentration-dependently increased (**Figure 6B**).

Angiogenesis is closely related to tumor metastasis and invasion. The migration and invasion ability of vascular endothelial cells is an important factor affecting neovascularization. Next, we investigated the effects of ZDQ-0620 treatment on HUVEC cell migration and invasion. Compared with control, the migration ability of HUVEC cells decreased gradually with the increase of ZDQ-0620 concentration (**Figures 6C, D**). Similarly, as shown in **Figure 6D**, ZDQ-0620 also inhibited the invasion of HUVEC cells in a concentration-dependent manner. These results indicate that ZDQ-0620 can significantly inhibit the migration and invasion ability of HUVEC cells *in vitro*, thus inhibit angiogenesis.

The interaction between tumor cells and vascular endothelial cells can play a synergistic role and promote the occurrence and progression of tumor. To investigate the effect of ZDQ-0620 on the interaction between HCT116 and HUVEC, the transwell chamber was used to verify the cell co-culture interaction. HUVEC was inoculated into the upper layer of transwell, and HCT116 was inoculated into the lower layer. As shown in **Figure 6E**, the presence of HCT116 cells in the lower layer significantly improved the HUVEC metastasis and invasion ability in the upper compartment. The migration and invasion levels were significantly inhibited with the addition of ZDQ-0620. Similarly, HCT116 was inoculated into the upper layer of transwell and HUVEC into the lower layer. Compared with the uninoculated cells in the lower layer, the migration and invasion of HCT116 in the upper layer were significantly increased after inoculation with HUVEC. The migration and invasion ability were also significantly inhibited with the addition of ZDQ-0620 (**Figure 6F**).

### ZDQ-0620 Inhibits Endothelial Cell Tube Formation and Vasculogenic Mimicry (VM) of HCT116

**Figure 7:**
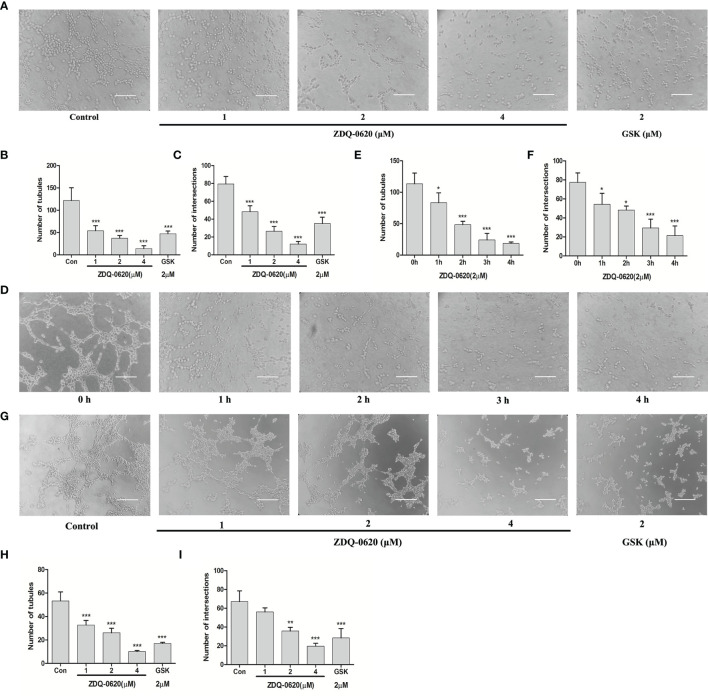
ZDQ-0620 inhibits endothelial cell tube formation and vasculogenic mimicry (VM) of HCT116. **(A, D)** ZDQ-0620 inhibited tube formation in HUVECs. Scale bar, 100 μm. **(B, C, E, F)** Histogram showing the average number of cells in the five random areas on each filter under each condition. **(G)** Decrease of VM formation in HCT116 cells after ZDQ-0620 treatment, Scale bar =100 µm. **(H, I)** The number of microtubules and the number of microtubule nodes decreased after treatment with different concentrations of ZDQ-0620. Each value is the mean (± SD) from triplicate samples; *p < 0.05, **p < 0.01, ***p < 0.001 *vs*. the control. One-way analysis of variance followed by Tukey’s *post hoc* multiple-comparisons test.

Endothelial differentiation (tube formation) is necessary for angiogenesis. ZDQ-0620 had little effect on the survival of HUVEC cells (**Figure 1C**), suggesting that ZDQ-0620 had low cytotoxicity to HUVEC. After treatment with ZDQ-0620, the tube formation of HUVEC cells was investigated, and it was observed that ZDQ-0620 significantly inhibited the catheterization of HUVEC cells in a concentration- (**Figures 7A–C**) and time-dependent manner (**Figures 7D–F**), ZDQ-0620 reduces the number of tubules and nodes.

The formation of VM leads to poor prognosis and promotes tumor hematogenous metastasis. Next, the ability of HCT116 to simulate microtubule formation in endothelial cells was verified, and the effect of ZDQ-0620 on the angiogenic mimicring-inhibiting activity of HCT116 was investigated. As shown in **Figure 7G**, HCT116 formed obvious microtubule connections. ZDQ-0620 could significantly inhibit the formation of angiogenic mimicry in a concentration-dependent manner (**Figures 7H, I**). Among them, the number of tubules and nodes in angiogenic mimicry concentration-dependently decreased after ZDQ-0620 treatment. In conclusion, ZDQ-0620 can inhibit the formation of angiogenic mimicry in HCT116 and play an indirect anti-angiogenic role.

### ZDQ-0620 Suppresses VEGF-Induced Neovascularization of Rat Aortic Ring and CAM

**Figure 8:**
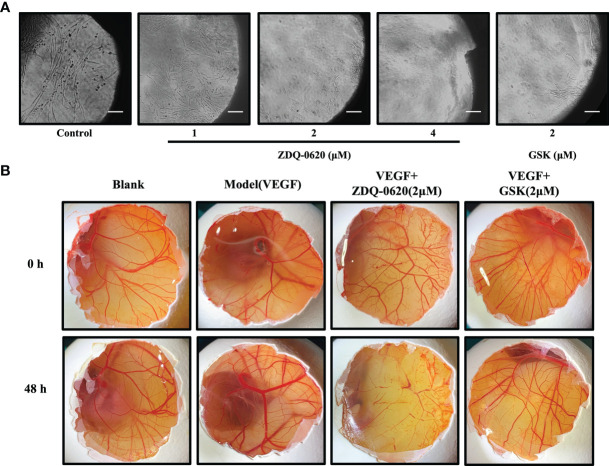
ZDQ-0620 inhibits angiogenesis *ex vivo* and *in vivo*. **(A)** ZDQ-0620 inhibited microvascular sprouting in rat aortic rings; scale bar, 100 μm. **(B)** Chicken eggs were treated with vehicle, VEGF (50 ng/mL), or VEGF+ZDQ-0620 or GSK2126458. Representative images from separate experiments are shown. All experiments were repeated three times.

In order to further verify the anti-angiogenic activity of ZDQ-0620, we first utilized rat aortic ring sprouting in *ex-vivo*. According to the results in **Figure 8A**, VEGF group (control) significantly stimulated the germination of rat aortic ring vessels and formed tubules covering the aortic ring. However, ZDQ-0620 could significantly inhibit VEGF-induced vascular germination in a concentration-dependent manner.

In addition, chicken embryo chorioallantoic membrane (CAM) was used to simulate angiogenesis *in vivo*. As shown in **Figure 8B**, compared with the blank group, VEGF could accelerate the new generation and growth of allantoic membrane blood vessels. VEGF group had more branches of vascular network, thicker vascular diameter and formed a dense vascular network after 48h treatment. However, ZDQ-0620 could significantly inhibit VEGF induction with sparse distribution of vascular network and low bifurcation degree, and its inhibitory activity was significantly stronger than that of positive drug GSK2126458. It is suggested that ZDQ-0620 can inhibit the angiogenesis *in vivo*. These data validate that the effects of ZDQ-0620 on the initiation stage of angiogenesis can be translated into the final suppression of microvessel formation.

## Discussion

The PI3K/AKT/mTOR pathway plays a critical role in the occurrence and progression of colorectal cancer (19, 20). Excessive activation of various receptor tyrosine kinases (such as insulin-like growth factor (IGF) and epithelial growth factor receptor (EGFR)) can also promote abnormal activation of this pathway (21). In addition, genes in the PI3K/AKT/mTOR pathway change most frequently in human cancers (22). Due to these somatic mutations, abnormal activation of this pathway is associated with cell transformation, tumorigenesis, angiogenesis and cancer progression (10, 23). Therefore, the PI3K/AKT/mTOR pathway has been a popular target for antitumor drug research in the past decades (24). However, the optimal treatment strategy for this pathway has not been established in CRC.

In this study, we investigated the anti-cancer effects of 4-aminoquinazoline derivative ZDQ-0620, and further elucidated its mechanism as a novel PI3K inhibitor. For the first time, we reported that ZDQ-0620 has excellent activity on proliferation, migration, invasion and angiogenesis of CRC cells by blocking the PI3K/AKT/mTOR pathway. And the activity of ZDQ-0620 was significantly better than that of the positive drug GSK2126458, a PI3K inhibitor.

Since the regulation of PI3K/AKT/mTOR pathway can promote cell survival and proliferation, our study shows that the reduction of HCT116 cell proliferation by ZDQ-0620 seems to be related to the PI3K/AKT/mTOR pathway. So we first used western blot assay to identified that ZDQ-0620 remarkably suppressed the activation of AKT, mTOR, p70S6K, and 4EBP1 in CRC cancer cell HCT116, thus inhibited PI3K/AKT/mTOR pathway. Moreover, vitro molecular docking results indicated that ZDQ-0620 inhibited 50% PI3K subtype activity (IC50) below 152 nM dose. The IC50 inhibition rate of PI3Kα reached 0.5nM, which was significantly stronger than other subtypes, with a difference of about 1000 times. It is suggested that ZDQ-0620 may be a PI3Kα target inhibitor, rather than a generic PI3K inhibitor. So, CETSA was further used to evaluate the binding ability of PI3K and ZDQ-0620. By CETSA assay, both ZDQ-0620 and DMSO obtained apparent aggregation temperatures (TAGG) that could be compared and showed significant changes indicating that ZDQ-0620 binds to the target protein (25). Western blot results suggested that ZDQ-0620 could significantly improve the binding ability with PI3K target protein, and the binding strength was dose-dependently. CETSA results confirmed that the compound ZDQ-0620 may target PI3K. Meanwhile, the CETSA Melt Curve showed that the binding ability of ZDQ-0620 to PI3Kα was significantly stronger than that of ZDQ-0620 to PI3Kβ, confirmed that the compound ZDQ-0620 may target PI3Kα.

Apoptosis plays an important role in the process of tumor proliferation and is a key mechanism to inhibit the growth of cancer cells (26). At the same time, the molecular mechanisms of apoptosis has also been confirmed to play an important role in the anti-tumor effect of PI3K/AKT/mTOR pathway (26–28), by activating various apoptotic signals or inhibiting survival signals (29, 30). In this study, we demonstrated that ZDQ-0620 can induce the apoptosis of HCT-116 cells through G0/G1 cell-cycle arrest. Bcl-2 is a key pro-apoptotic regulatory factor and its overexpression is associated with CRC (31). In addition, the Bcl-2 associated death promoters Bax is important target substrates for AKT (32–34). Bax is a pro-apoptotic protein that can be transported to mitochondria following the induction of cell death (31). Meanwhile, an increase in the Bax/Bcl-2 ratio upregulates the levels of cleaved caspase-3, leading to PARP cleavage and, consequently, irreversible apoptosis. In the present study, we found that ZDQ-0620 induces HCT-116 cell apoptosis by suppressing the expression of cleaved Bcl-2 and promoting that of Bax, cleaved caspase-3/9, and PARP. This suggested that the mitochondrial pathway may be the main mechanism underlying ZDQ-0620-induced apoptosis in CRC. In the PI3K/AKT/mTOR signaling pathway, AKT activates mTOR, thereby accelerating the transcription of genes required for cell cycle progression through the regulation of the downstream targets, 4EBP1 and p70S6K (35–37). Herein, we confirmed that the antiproliferative activity of ZDQ-0620 was related to G0/G1 cell cycle arrest, an effect that is likely achieved through a reduction in the expression levels of PI3K/AKT/mTOR pathway-related proteins.

When activated, the PI3K/AKT/mTOR signaling pathway not only modulates the translation of proteins involved in cell transformation and proliferation, but is also involved in tumor metastasis, invasion (38), and angiogenesis (39). AKT plays an important role in tumor invasion and metastasis by positively regulating the expression of MMP-2 (40). MMP-2 subsequently enhances the migration of cancer cells by degrading the extracellular matrix while also promoting metastasis through the induction of angiogenesis (41). In angiogenesis, the PI3K/AKT pathway is stimulated by multiple signals, including endothelial VEGF (42), and regulates multiple key steps through the phosphorylation of downstream target substrates, such as mTOR (43). In addition, the PI3K/AKT/mTOR pathway is associated with VEGF-induced endothelial signaling (43, 44). In Zang’s study (45), they identified that VEGF is a key factor in regulating angiogenesis and can be secreted from tumor cells. It was also confirmed that anti-tumor drugs inhibited the interaction between cancer cells and HUVECs by inhibiting VEGF. In addition, VEGF expression is regulated by NF-κB and PI3K/AKT signaling pathways; there is increasing evidence that PI3K pathway is involved in VEGFA dysregulation (46–49). The activation of endothelial PI3K/AKT/mTOR pathway signals can promote the survival of cultured microtubules *in vitro* (44) and tumor blood vessels *in vivo* (50, 51). Our results confirmed that ZDQ-0620 inhibits the migration, invasion, and angiogenesis of CRC cells, which may be related to a decrease in AKT phosphorylation levels or inhibit the secretion of chemokines, such as VEGF. These observations indicate that inhibition of the PI3K/AKT pathway may be the key mechanism underlying the anti-cancer effects of ZDQ-0620.

In conclusion, ZDQ-0620 was identified as being a PI3K target inhibitor, and displayed excellent anti-CRC activity, including the inhibition of cell proliferation, migration, invasion, protein synthesis, and angiogenesis, *via* the blocking of the PI3K/AKT/mTOR signaling pathway both *in vitro* and *in vivo*. Additionally, ZDQ-0620 was found to induce G0/G1 cell cycle arrest and mediate cell apoptosis through the mitochondrial pathway. Combined, these findings indicated that ZDQ-0620 has potential as a novel anti-cancer drug targeting the PI3K/AKT/mTOR pathway in CRC to inhibit tumor initiation and progression.

## Data Availability Statement

The raw data supporting the conclusions of this article will be made available by the authors, without undue reservation.

## Ethics Statement

The animal study was reviewed and approved by Ethics Committee for Animal Experiments of Shenyang Pharmaceutical University.

## Author Contributions

QZ and HD for supervision and funding acquisition. XQ for conceptualization, methodology, validation, formal analysis, writing - original draft, writing - review & editing and project administration. ML for investigation and resources. Others for editing and proofread. All authors read and approved the final manuscript.

## Funding

This research was supported by Liaoning Natural Fund Guidance Plan (Number: 2019-ZD-0446).

## Conflict of Interest

The authors declare that the research was conducted in the absence of any commercial or financial relationships that could be construed as a potential conflict of interest.

## Publisher’s Note

All claims expressed in this article are solely those of the authors and do not necessarily represent those of their affiliated organizations, or those of the publisher, the editors and the reviewers. Any product that may be evaluated in this article, or claim that may be made by its manufacturer, is not guaranteed or endorsed by the publisher.
